# The Role of Reverse Shoulder Arthroplasty in Elderly Trauma: A Systematic Review

**DOI:** 10.7759/cureus.8180

**Published:** 2020-05-18

**Authors:** Kawaljit Dhaliwal, Zuhaib Y Shahid, Baseem Choudhry, Craig Zhao

**Affiliations:** 1 Trauma and Orthopedics, Maidstone and Tunbridge Wells NHS Trust, Tunbridge Wells, GBR

**Keywords:** reverse shoulder arthroplasty, shoulder/ elbow arthroplasty

## Abstract

Objectives: The primary objective of this systematic review was to evaluate pain relief and shoulder functional outcome following reverse shoulder arthroplasty for three- and four-part proximal humerus fractures in patients over the age of 60 years. The secondary objective was to assess the clinical end radiological complications following this procedure for this indication.

Methods: Studies were identified using a MEDLINE search for relevant articles on 20th May 2019. The key terms ‘reverse shoulder arthroplasty’ and ‘proximal humerus fracture’ were used.

Results: Five retrospective case-series fully met the eligibility criteria. No randomized controlled trials or meta-analyses were found. All of the studies agreed that reverse shoulder arthroplasty was able to offer good pain relief, function end range of forward flexion (FF), and abduction (Abd.). Restrictions in shoulder rotation have to be fully addressed. The rate of major complications, reduction in functional outcome, and development of scapular notching with time was a concern.

Conclusions: Reverse shoulder arthroplasty for comminuted proximal humerus fractures has increased over the past several years, yet the published data evaluating the surgical outcome is limited. Large well-designed prospective randomized controlled trials are needed for comparing the various treatment options, in order to ensure that these patients receive the best treatment available.

## Introduction and background

Proximal humerus fractures are the second most common upper extremity fracture and the third most common fracture affecting patients over the age of 65, following hip and distal radius fractures [1]. They usually result from simple falls onto the outstretched limb or the shoulder itself. These are usually osteoporotic fractures. As the population ages and life expectancy continues to increase, these injuries are becoming increasingly common. The majority are two-part fractures with minimal displacement and are successfully managed conservatively with early rehabilitation. However, the optimal management of complex three- and four-part displaced fractures of the proximal humerus in elderly patients with poor bone quality remains controversial. These pose a challenge to every orthopedic surgeon as there is a wide variety of surgical options available as well as nonoperative management being advocated in some centers.

Locking plates may provide adequate angular stability in the face of axial load, promote revascularization and allow early rehabilitation, but have uncertain results in patients with severe osteoporosis and comminution. Their clinical benefit in the treatment of two-part fractures has been established. However, the overall complication rate when used for more complex fractures with severe osteoporosis is substantial. The risk of osteonecrosis, loss of fixation, varus subsidence, screw perforation of the humeral head, and malunion are challenging complications that result in postoperative pain and reduced mobility. This may lead to an unsatisfactory surgical outcome [2]. Hence, for comminuted proximal humerus fractures, prosthetic replacement of the humeral head appears to be justified, particularly if there is associated dislocation of the glenohumeral joint, head-splitting fractures, metaphyseal hinge attached to the articular surface of <2 mm and severe osteoporosis, which may all increase the risk of avascular necrosis with osteosynthesis [3].

Shoulder hemiarthroplasty is known to reliably offer patients good relief of pain. However, an unsolved problem with hemiarthroplasty for these fractures is the significant functional deficits in the range of motion (ROM) and activities of daily living. Constant scores are typically in the mid 50s to low 60s [4]. Anjum and Butt reported that the ROM in elderly patients was disappointing. At a mean follow-up of 33 months the mean constant score was 47.5 [5]. Demirhan et al. [6] showed that tuberosity migration, nonunion, malunion or resorption and rotator cuff dysfunction resulted in poor clinical outcomes. Rotator cuff injury is seen in 28% of the patients between 60 and 70 years, in 50% of the patients between 70 and 80 years, and in 80% of the patients older than 80 years [7].

The reverse shoulder arthroplasty, which was initially described by Boileau et al. was designed specifically for the treatment of rotator cuff arthropathy [8]. Mid-term results using this prosthesis for rotator cuff arthropathy have been described with good functional outcomes [9-11]. The prosthesis design converts the glenoid into a spherical head and the head of the humerus into a socket, thus providing a stable fulcrum for glenohumeral joints with deficiency of the rotator cuff. The reverse shoulder arthroplasty relies on the deltoid muscle and hence the integrity of the axillary nerve. With reverse geometry, the fixed center of rotation is located more distal and medial which leads to an increase of the deltoid muscle efficiency by 25%-30%.

Due to the reverse design, the prosthesis is especially suitable for patients who have rotator cuff tears. The reverse shoulder concept does not rely on the integrity of the rotator cuff or the tuberosities to which they attach. As a result of this potential benefit, the reverse shoulder arthroplasty has recently been introduced as an alternative treatment option for elderly patients with comminuted proximal humerus fractures.

Objectives

(1) The primary objective of this systematic review was to evaluate pain relief and shoulder functional outcome following reverse shoulder arthroplasty for three- and four-part proximal humerus fractures in patients over the age of 60 years.

(2) The secondary objective was to assess clinical and radiological complications following this procedure for this indication.

## Review

Materials and methods

Study Identification

The databases used for this study were MEDLINE(Ovid) and the Cochrane library. Studies were identified on 20th May 2019.

The following searches were used:

1. MEDLINE searches: Using the key terms ‘reverse shoulder’ OR ‘inverse shoulder’ OR ‘delta shoulder’ arthroplasty AND combined with ‘proximal humerus fracture’ OR ‘shoulder fracture’.

2. Cochrane database searches: Using terms ‘reverse shoulder arthroplasty’ and ‘proximal humerus fracture’.

Limits were applied for articles in English, human studies, all adults, randomized controlled trials, meta-analyses, clinical trials, controlled clinical trials, comparative studies, journal articles, and multicenter studies to refine the search. Biomechanical studies, Cadaveric studies, editorials, letters, comments, case reports, and review articles were excluded from the limits search.

All citations were imported onto Endnote Web to remove duplicate studies. All titles and abstracts were reviewed and relevant full-text articles were obtained. Each article was then reviewed against the eligibility criteria to determine whether it would be included in this systematic review.

Eligibility Criteria

Articles that met the following criteria were identified:

Inclusion criteria:

1) The target population consisted of elderly adult patients (>60 years) with a three- or four-part proximal humerus fractures.

2) The intervention was primary treatment of a comminuted proximal humerus fracture with a reverse shoulder arthroplasty.

3) Outcome measures for pain relief, range of movement/function, complications, and radiological assessment needed to be included in the study. 

4) All study designs from Levels I-IV were eligible.

Exclusion criteria:

1) Small studies with sample size of less than 20 patients treated with reverse shoulder arthroplasty were excluded [12].

2) Articles not assessing functional outcomes [13].

3) Studies with one- or two-part proximal humerus fractures.

4) Articles not in English.

Data extraction

For each eligible study, data were extracted for the study design, aim of the study, demographic data (age, sex), patient selection criteria, group sizes, intervention protocol, duration of the follow-up, surgical techniques, numbers lost to follow-up, potential bias, and study outcome measures. Outcome measures for pain, shoulder function, complications, and radiographic outcomes were collected.

Results

The literature search identified 47 potentially relevant citations when the limits were applied. All citations were from MEDLINE(Ovid). No citations were identified on the Cochrane database. There have been no randomized controlled trials or meta-analyses published on the use of reverse shoulder arthroplasty for proximal humerus fractures.

After reviewing the title and abstracts, 13 articles were found relevant. Seven articles met the inclusion criteria. Five studies fully met the eligibility criteria and were critically appraised. Four studies were retrospective case-series (Level 4 evidence) with one prospective case series (Level 4 evidence).

**Figure 1:**
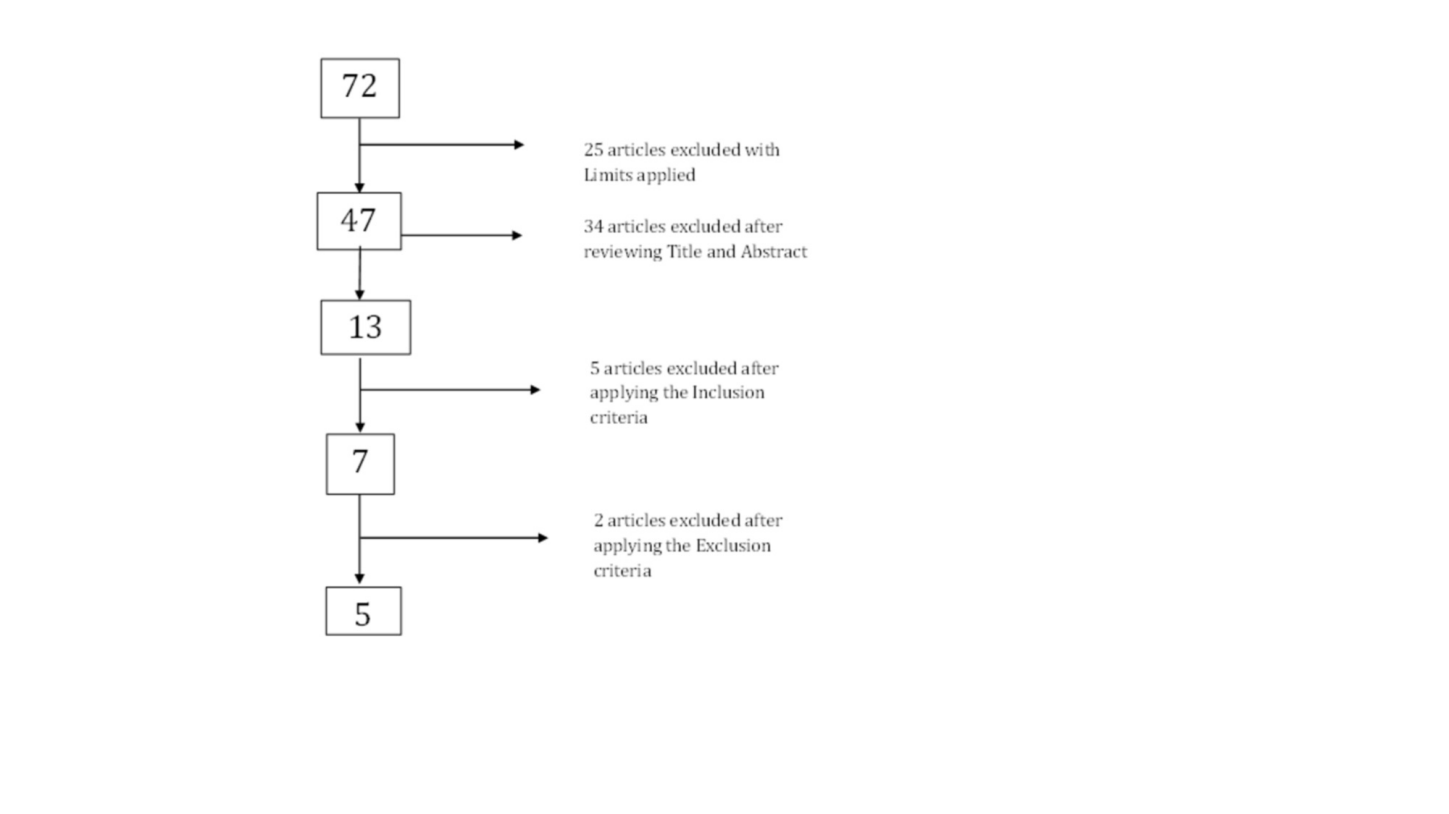
Flow diagram of literature search: MEDLINE (Ovid) search using key terms: ‘reverse shoulder’ OR ‘inverse shoulder’ OR ‘delta shoulder’ arthroplasty AND combined with ‘proximal humerus fracture’ OR ‘shoulder fracture’.

A flow diagram of this reproducible literature search is shown in Figure 1.

Critical appraisal

Study 1: Bufquin et al. (2007). ‘Reverse shoulder arthroplasty for the treatment of three- and four-part fractures of the proximal humerus in the elderly’ [14].

This is a retrospective case-series assessing the clinical outcome of reverse shoulder arthroplasty in elderly patients with comminuted proximal humerus fractures. All patients over the age of 65 with three- or four-part proximal humerus fractures. Patients with an active infection, axillary nerve palsy, a deficient deltoid muscle, or a bone tumor were excluded [9].

Patient demographics are summarized in Table 1. Five patients had displaced three-part fractures, 38 had four-part fractures, of which 12 had dislocations. Mean follow-up was 22 months (6-58 months). The authors fail to mention the co-morbidities of the patients selected for this procedure, which raises the issue of possible selectional bias.

**Table 1:** Study design and patient demographics. #, fracture; x, multiplied; OTA, Orthopaedic Trauma Association; RC tears, rotator cuff tears; DM, diabetes mellitus; ETOH, alcohol excess; PVD, peripheral vascular disease; BP, blood pressure; COPD, chronic obstructive pulmonary disease; ASES, American Shoulder and Elbow Surgeons; DASH, disabilities of the arm, shoulder, and hand; SF, San Francisco.

Study	Bufquin et al. (2007) [14]	Klein et al. (2008) [15]	Cazeneuve and Cristofari (2010) [16]	Villodre-Jimenez et al. (2016) [17]	Grubhofer et al. (2016) [18]
Study design	Retrospective case-series	Retrospective case-series (prospectively collected)	Retrospective case-series	Prospective case-series	Retrospective case-series
Level of evidence	Level IV	Level IV	Level IV	Level IV	Level IV
Year of investigation	Jan 2000–Jan 2005	July 2002–Dec 2004	Feb 1993–Oct 2009	December 2008–June 2014	October 2005–October 2013
Number of patients	43	20	47	30	73
Follow-up numbers	41 (95%)	20 (100%)	36 (77%)	30 (100%)	51 (70%, 52 shoulders)
Follow-up (months)	22 (range 6–58)	33.3 (range 24–52)	79.2 (range 12–192)	34.5 (SD = 19.3)	35 (range 12–90)
Mean age at operation (years)	78 (range 65–97)	75 (range 67–85)	75 (range 58–92)	74.9 (SD = 6.3)	77 (range 58–89)
Sex – Female:Male	41:2	14:6	34:2	26:4	45:6
Dominant arm	26 (40%)	13 Right: 7 Left	16 Right : 20 Left	Not stated	31 Right:13 Left
Fracture types	Displaced 3 part – 3 and 4-part – 38 # Dislocations – 12	OTA Type B2 – 5 Type C2 – 7 Type C3 – 8 (Based on CT)	3 and 4-part – 26 with dislocations - 10	3-part – 27% 4-part – 73%	Head-splitting fracture – 10 3-part – 4 4-part – 38
Co-morbidities	Not stated	Cardiac x8 PVD x4 BP x 15 COPD x2 DM x3 Thyroid disease x3 Renal disease x2	Known RC tears – x6 DM – x5 Morbidly obese x4 Etoh x3	Not stated	Not stated
Mean time from injury to operation	<15 days	10.05 days (range 3–41)	Not stated	Not stated	5 days (range 0–16)
Surgical intervention	Four different surgeons no. of centres – not stated Approach: Superolateral (n=20) Deltopectoral (n=23) Delta reverse prosthesis (Depuy, Leeds, UK)	No. of surgeons – not stated Single center Approach: All Anterolateral Delta III reverse prosthesis (Depuy)	No. of surgeons – not stated. Single center Approach: All Anterolateral Grammont reverse prosthesis	Two different surgeons Single center Approach: All Deltopectoral Lima SMR model	No. of surgeons – not stated two centers Approach: not stated Zimmer Reverse Anatomical Shoulder System
Outcome measures used	Constant Score Modified Constant ASES score DASH Radiological – (loosening and notching) Complications	Constant Score Modified ASES score DASH SF-36 Radiological Complications	Constant Score Radiological Complications	Abbreviated Constant scale QuickDASH UCLA scale Radiological – (Loosening, notching, greater tuberosity position and arm length) Complications	Constant Score Subjective Shoulder Value Age and gender matched relative CS Pain level Patient’s outcome satisfaction Range of motion Radiological – (loosening, notching, greater tuberosity position) Complications

Operations were performed by four different surgeons, all using the same implant (Reverse Delta prosthesis, DePuy, Leeds, UK). All operations were carried out within 15 days of the injury. Two different approaches were used. Superolateral was used for the first 20 patients, however, the authors decided to convert to the deltopectoral approach for the remaining 23 to avoid dividing the deltoid muscle. The operation was well described with the supraspinatus tendon and long head of the biceps divided, if present. Half of the stems were inserted in retroversion and remainder in neutral to increase internal rotation. The conversion in the type of approach, as well as a change in the stem version, although clinically pragmatic, does add a confounder into the intervention that may have independently affected the outcome. The postoperative rehabilitation was consistent for all patients and involved immobilization for two days followed by active physiotherapy. The authors did not provide any details of the physiotherapy received.

The primary and secondary outcome measures were not clearly stated. However, a comprehensive assessment was carried out at six monthly follow-ups by one independent assessor. The authors have used validated, patient-reported and physician-reported, disease-specific functional outcome measures to record results. Constant and Murley, American Shoulder & Elbow Surgeons (ASES), Disabilities of the Arm [14]. Shoulder and Hand (DASH) scores and the modified Constant score (calculated as a percentage of the normal value relative to gender and age) were all used to assess pain and functional outcome. Radiological assessment for loosening, scapular notching, as well as clinical complications was assessed. Although the authors have attempted to reduce reporting bias by using an independent assessor, they have not provided details of the assessor and have introduced some intra-observer error by using one assessor only.

Postoperative results were described in reasonable detail and are summarized in Table 2. Two patients were excluded from the assessment as they had died from unrelated causes. Hence results were available for 41 patients at follow-up (4.6% lost to follow-up). The authors estimated preoperative shoulder movement and outcome scores based on the patients’ contralateral shoulder. Range of movement and outcome scores were presented in a table format allowing easy comparison. After the operation, none of the ranges of movement matched those of the contralateral shoulder. The mean active forward flexion (FF) was 97°, abduction (Abd.) was 86°, and external rotation was 30°. The authors failed to mention the range of internal rotation. The mean Constant score, the mean modified Constant score, and the ASES score were all less than in the opposite shoulder. The mean Constant score was 44 compared to 69 points in the contralateral limb (note: higher the Constant score = better the outcome).

The authors describe their 12 major complications in detail. These include one intra-operative glenoid fracture, which was treated immediately by a revision baseplate. Five patients reported neurological complications (three median, one axillary, one ulnar nerve), which eventually recovered. One patient sustained an acromion fracture 12 months postoperatively, which healed uneventfully. Three patients developed reflex sympathetic dystrophy, which again resolved spontaneously. One patient further had a nontraumatic anterior dislocation at six weeks, but declined further surgery after reduction. One patient required re-operation at 17 months due to separation of the anterior deltoid muscular flap resulting from a supero-lateral approach.

Three patients had no radiographs; therefore, radiological evaluation was only possible in 40 patients (93%). Radiological assessment reported no loosening of either component. In 36 shoulders in which the tuberosities had been fixed, secondary displacement occurred in 19 (53%) leading to malunion in 5 (13.8%) and nonunion in 14 (38.8%). Scapular notching was observed in 10 shoulders (25%), with only one at Sirveaux grade 3. The authors report that notching was generally noted within the first year and that this did not progress with follow-up. Heterotopic ossification was noted in 36 shoulders (90%), but the effect on the functional outcome appeared to be limited.

The authors state that there is no correlation between the grade of notching and the angle of inclination of the glenoid component (Spearman correlation coefficient r = 0.23, p = 0.20). The authors also mentioned that clinical results were not influenced by the type of approach or the healing of the tuberosities. However, the authors fail to demonstrate this with the results presented, as they have not provided subgroup analysis outcomes.

The authors report that patients older than 75 years had lower Constant scores (Kruskal-Wallis p = 0.06), external rotation was greater when the greater tuberosity had healed anatomically (Kruskal-Wallis p = 0.07), and patients with a low shoulder center had better results (Kruskal-Wallis p = 0.09), although these differences were not statistically significant.

On interpreting the results, the rate of loss of patients in this study was low and lower than that seen in other series dealing with fractures of the proximal humerus; however, the follow-up was short. Major complication rates observed were high. Relief from pain and improvements in anterior elevation and Abd. were the most noted improvement in those who did not experience a complication.

Study 2: Klein et al. (2008). ‘Treatment of comminuted fractures of the proximal humerus in elderly patients with the Delta III reverse shoulder prosthesis’ [15].

The aim of this study was to assess the clinical and radiological outcomes of the Delta III reverse shoulder prosthesis implanted into patients over the age of 65 years with comminuted proximal humerus fractures at a mean follow-up of 33.3 months. This is a retrospective case-series performed over a 2 ½ -year period in a single Level II trauma center. Patients with local or systemic infections, severe glenoid deformity (based on Walsh classification - Type B2 or C), systemic muscle disease, vascular or nerve diseases, dementia, axillary nerve palsy, or an American Society of Anesthesiologists score (ASA) >3 were excluded [15].

Patient demographics are summarized in Table 1. All patients underwent a preoperative CT scan to grade the fracture and glenoid. Five patients had an OTAAQ type B2, seven with a type C2 and eight with a type C3 fracture pattern.

The intervention was a Delta III reverse prosthesis (DePuy Orthopedics, Leeds, UK) inserted through an antero-lateral approach. The tuberosities were removed without reattachment of the rotator cuff muscles in all patients. The cemented stem was inserted in 15° retroversion and the glenoid component slightly inferior to the glenoid center. The authors have made effects to reduce confounding factors by keeping a consistent surgical technique in all patients. The postoperative regime was described in detail, which involved the use of an Abd. pillow for the first four weeks and with a passive range of movement commencing on day 2. Anterior elevation and Abd. were limited to 90 degrees for the first four weeks.

At follow-up, validated outcome measures used included the Constant score, modified ASES score, DASH score, and SF-36 (36-item short-form health survey) to evaluate the clinical and functional outcomes [15]. Radiological assessment was performed using anteroposterior (AP) and axillary lateral views. Radiological evidence of loosening of the stem included progression of radiolucent lines, widening of the humeral canal, osteolysis, and migration of the stem. Radiographs were also evaluated for inferior notching at the glenoid by Nerot’s classification [15].

Results of the data were prospectively collected and summarized in Table 2. No patients were lost to follow-up. Operations were performed at a mean 10 days after trauma. Duration of surgery was 50-120 min. Postoperative scores for range of movement were 123° for mean FF and 113° for mean Abd. Mean internal rotation was possible up to L4, and the external rotation was 25°. The mean Constant score was 67.85 (range 47-98) points. Radiological analysis showed no evidence of loosening of the humeral stem or the base plate. No osteolysis, migration, or radiolucent lines were reported. One patient had signs of inferior notching at Nerot grade 1. The authors have not mentioned the number of assessors, who were involved in the assessments or how the ROM was assessed. It is therefore difficult to comment on issues relating to blinding of assessors, observer bias, intra- and inter-observer error, and measurement bias in the reporting of results.

Major complications occurred in three patients (15%). One patient was dislocated twice and underwent closed reduction under general anesthesia. Two patients developed early deep infections and required four washouts each and six weeks of antibiotics postoperatively. No neurological complications were reported.

The limitations of this study include a lack of a control group, a small number of patients, relatively short follow-up period, as well as a lack of detail on how the results were recorded. The authors conclude that besides good pain relief, the reverse shoulder offers an excellent functional outcome in terms of anterior elevation and Abd. Internal rotation and external rotation are still limited. The authors hypothesize that rotation may be improved by reattachment of the tuberosities, but this was not demonstrated in this study.

Study 3: Cazeneuve and Cristofari (2010). ‘The reverse shoulder prosthesis in the treatment of fractures of the proximal humerus in the elderly’ [16].

This is a retrospective case-series of patients that underwent a reverse shoulder arthroplasty for a complex proximal humerus fracture. The aim of the study was to report the clinical and radiological outcomes at a mean 6.6 years follow-up (range 1-16 years). The eligibility criteria for the study were three- or four-part fractures in elderly patients with osteoporosis. Pathological fractures were excluded.

The characteristics of the study population are represented in Table 1. The mean age at operation was 75 years. Some 26 patients had three- and four-part fractures; a further 10 had fracture dislocations. The population consisted of five patients with diabetes, four morbid obesity, and three with a history of alcohol abuse. It appears the authors have made efforts to avoid excluding patients based on their past medical history and possible selectional bias, however, this cohort is at higher risk and therefore could negatively impact the outcomes.

The surgical intervention involved inserting the Grammont reverse prosthesis using an anterolateral approach in all patients, in a single unit. Remnants of the tuberosities were excised. The authors felt this would prevent the limitation of adduction and possible instability of the humeral component. The humeral component was inserted in 10°-20° of retroversion, except in one case where it was inserted in 10° anteversion to try to increase the internal rotation. Low viscosity cement was used. The long head of the biceps tendon was sutured to the lateral fin of the humeral component. Postoperative information was sparse, but involved immobilization in a sling followed by active physiotherapy as tolerated.

Primary and secondary outcomes were not clearly stated. However, the outcome was assessed using the Constant score, which is a validated, patient-reported and physician-reported, disease-specific functional outcome measure, and compared to the contralateral side. Radiological assessment for loosening and impingement, and clinical complications were also assessed.

Some 47 patients had undergone a reverse shoulder arthroplasty. Some 11 patients were lost to follow-up (23%), nine had died from unrelated causes, and two had left the area. Some 36 patients were therefore available for follow-up assessment. Clinical outcome results were reported with a breakdown of the Constant score into subjective and objective assessments (results summarized in Table 2). The mean Constant score was 53 points at a mean follow-up of six years. This represented 67% of the mean score for the uninjured contralateral shoulder side (79 points). The mean modified Constant score was 69.3 points, after adjusted for age and sex. The Constant pain score was 12 point (corresponds to VAS of 3). The Constant range of movement scores was a mean 7.5 points for FF (120° > FF < 150°), 6.5 points for Abd. (120° < Abd. < 150°), 1 point (1 - 2 points) for internal rotation, and 1 point (1-4 points) for external rotation. The authors fail to clearly define the mean range of movements in their study and therefore we have extrapolated the range from the Constant score to allow comparison with other studies in this systematic review. This study also provides no information on the number of assessors, who the assessors were, and how the ROM was recorded. It is therefore difficult to comment on observer bias, intra- and inter-observer error, and measurement bias. The authors provide a table comparing the outcome at year one postoperation against the last follow-up outcome. There is a subtle decrease in all components of the Constant score with the raw Constant score reducing from 55 points (range 20-84) to 53 points (range 20-80) at last assessment. 

Radiological assessment showed 63% of patients with some glenoid loosening, however, only one case of aseptic loosening of the glenoid base-plate occurred at 12 years. Scapular notching was noted in 19 patients (25%) according to the Nerot classification. The amount of notching was not stated or whether the patients were symptomatic with this.

Some seven clinical complications were reported (19.4%). One anterior dislocation occurred in one patient with an anteverted humeral stem, which required revision surgery. Three patients had superior dislocations due to impingement on the remnants of the tuberosities, which were subsequently removed. The principal complication was dislocation (11%), which is preventable with improved surgical technique. Two patients developed a complex sympathetic dystrophy, which had resolved in both patients by nine months. One diabetic patient developed an early postoperative Acinetobacter infection, which had resolved after irrigation and debridement.

The authors emphasize the importance of surgical technique in performing this procedure, advise removal of the tuberosities completely and avoidance of anteversion of the humeral component, both of which may lead to dislocation. Based on the results represented and the length of time the patients were followed-up, we concur with the authors in that the reduction in functional outcome and mean Constant scores with time, and the further development of scapular notching was a concern.

Study 4: Villodre-Jimenez et al. (2016). ‘Reverse shoulder arthroplasty in 3 and 4 part proximal humeral fractures in patients aged more than 65 years: results and complications’ [17].

The objective of this study was to evaluate the functional results in patients over 65 years old with three- and four-part complex proximal humeral fractures treated with reverse shoulder arthroplasty, at a mean follow up of 34.5 months. This was a prospective study performed over 5½ year period in a single center by two different surgeons.

Patient demographics are summarized in Table 1. Some 30 patients were included in the study with the mean age at the time of surgery being 74.9 years. Some 27% were three-part and 73% were four-part fractures according to Neer’s classification system [17].

All patients had a Lima SMR Reverse shoulder arthroplasty with a cemented stem (retroverted to 30°) through deltopectoral approach. The tuberosities were reconstructed in all cases with nonresorbable material. Mean surgical time was 90.2 min. Postoperatively, shoulder was fixed in a sling for three weeks, after which pendular exercises and passive Abd. and antepulsion (up to 90°) began.

All patients were followed up at one month, six months, and annually. Abbreviated Constant scale, QuickDASH, and UCLA scale were used to evaluate the clinical outcomes. Radiological assessments were performed looking for scapular notching, displacement/non-union of the tuberosity, and also the arm length following surgery [17].

Results of the data were prospectively collected and summarized in Table 2. No patients were lost to follow-up. Postoperative range of movement was 124° for mean FF and 95° for mean Abd. 13° was the mean external rotation achieved, and L5 was the anatomic region most frequently reached on internal rotation. Radiological assessment showed scapular notching in 14 patients (46%) and tuberosity displacement or nonunion in 33% of patients. The study showed no relationship between the presence of notching and functional outcome and pain. The group with anatomical union of tuberosity presented better mobility outcomes, although the differences were not statistically significant. The increase in mean length of operated limb was 13.4 mm. Group with lengthening of less than 20 mm achieved better scores, which was statistically significant.

**Table 2:** Results – clinical and radiological outcomes. ROM, range of motion; FF, forward flexion; Abd., abduction; LHB, long head of biceps; RC, rotator cuff; VAS, Visual Analog Score; ASES, American Shoulder and Elbow Surgeons; ACS, acute coronary syndrome; CRPS, complex regional pain syndrome.

Study	Bufquin et al. (2007) [14]	Klein et al. (2008) [15]	Cazeneuve and Cristofari (2010) [16]	Villodre-Jimenez et al. (2016) [17]	Grubhofer et al. 2016 [18]
Technical details of operation	Supraspinatus/LHB tendon divided, if present Stem version: 50% retroversion (degrees – not stated) and 50% neutral Tuberosities repair attempted in all	15 ° stem retroversion Tuberosity removed without reattachment of RC muscles	LHB preserved Stem retroversion 10–20 ° . 1 stem = 10 ° anteversion Tuberosity remnants removed	30 ° stem retrovesion Cemented humeral stem fixation Tuberosity reconstructed in all cases	0–20 ° stem retroversion Decision to cement stem made intraoperatively Tuberosity reattached if possible (remnants resected in four cases)
Mean ROM	FF – 97 (35–160 ° ) Abd. – 86 ° (35–150) Ext. rotation in abd. – 30 ° (0–80) Int. rotation – not stated	FF – 123 ° (60–175 ° ) Abd. – 113 ° (60–180 ° ) Ext. rotation – 25 ° (10–35 ° ) Int. rotation – L4	Constant FF – 7.5 (5–9). Mean approx 120 ° Constant Abd. – 6.5 (4–9). Mean approx 120 ° Constant Ext. rotation – 1 (1–4) Constant Int. rotation – 1 (1–3)	FF – 124 ° (SD = 30.3) Abd. – 95 ° (SD = 34.7) Ext. rotation – 13 ° (SD = 28) Int .rotation – L5	FF – 118 ° (40–165 ° ) Abd. – 111 ° (40–165 ° ) Ext. rotation – 18 ° (0–65 ° ) Int. rotation – 5 ° (0–10 ° )
Mean outcome measure scores	Constant score – 44 (16–69) Modified Constant – 66% (25–97) Constant score for pain – 12.5 (5–15) = VAS 2.5 Constant score for activity – 10.9 (4–20) Constant score for ROM – 17.6 (2–34) Constant score for strength – 7.5 (2–12) ASES score – 9 (0–19) DASH score – 44 (0–92)	Constant score – 67.9 (47–98) Modified ASES – 68 (50–90) DASH – 46.85 (6–63) SF-30 – physical 38 (13–57) and mental 52.59 (32–73)	Constant score – 53 (20–80) Modified Constant score – 69.3 Constant score for pain – 12 (10–13) = VAS 3 Constant score for activity – 13 (8–17) Constant score for strength – 12 (10–15)	Abbrev. Constant – 49.1 (SD = 14.1) QuickDASH – 32.2 (SD = 19.2) UCLA scale – 27 (SD = 6.3)	Constant score – 62 (21–83) Relative Constant – 86% (30–100) Subjective Shoulder Value – 83% (30–100) Constant score for pain – 14 (5–15) Satisfaction – 35 Excellent, 13 Good, 4 Fair, 0 Dissatisfied
Radiological	No loosening of stem or glenoid component Tuberosity malunion – 5 (13.8%) Tuberosity nonunion 14 (38.1%) Scapular notching – 10 (25%). (1 Sirveaux grade 3)	No loosening of stem or glenoid component 1 Scapular notching (Nerot grade 1)	1 aseptic glenoid loosening at 12 years Glenoid loosening seen in 63%. Scapular notching – 19 (25% on Nerot. Grade not stated)	Tuberosity displcement / nonunion – 33% Increase in mean length of operated limb – 13.4 mm (SD = 9.3) Scapular notching – 14 (46%)	No loosening of stem or glenoid component Tuberosity displaced – 4 (8%) Tubersity resected (due to pluri-fragmentation) – 4 (8%) Scapular notching – 33 (63%)
Complications	N = 12 (29%) 1 Glenoid # 1 Acromion # 1 Ant dislocation 5 Neurological (3 median, 1 axillary, 1 ulnar nerve) 3 CRPS 1 Soft tissue complication-deltoid flap 36 Heterotopic ossification (90%)	N = 3 (15%) 1 Recurrent dislocation 2 Deep infections No neurological	N = 7 (19.4%) 1 Ant dislocation (from the one anteverted stem) 3 Sup dislocations 1 deep infection 2 CRPS	N = 4 (13.3%) 2 Intraoperative fracture of humerus 1 Periprosthetic fracture 1 Deep infection	Revision rate stated only – 5% (4/74) 1 Post operative hematoma 1 Periprosthetic humeral shaft fracture 2 Deep infections 1 Death from ACS, 4 days post uneventful surgery

Four complications were reported. Two patients sustained an intraoperative humeral fracture, who were treated using cerclages and humeral head graft. The same patient sustained three postoperative periprosthetic fractures, after three episodes of trauma. The first two occasions were treated with a plate, cerclages, and structural allograft. Long stem replacement was opted for the third episode as the fracture was distal to the tip of the stem.

Some limitations of this study include a lack of control group for comparison, small number of patients with a relatively short follow-up period. It is also possible that despite agreed methods for obtaining plain films, some measurements may have been affected by radiological views. The author concludes that total reverse arthroplasty is a valid procedure in treating complex proximal humerus fracture in the elderly population above 65 years of age, with predictable functional outcome and low complication rate. They emphasize the importance of reconstructing tuberosities, and avoiding increasing the limb length by more than 2 cm.

Study 5: Grubhofer et al. (2016). ‘ Reverse total shoulder arthroplasty (RTSA) for acute head-splitting, 3- and 4-part fractures of the proximal humerus in the elderly’ [18].

This is a retrospective case-series to assess the outcome of reverse total shoulder arthroplasty for acute proximal humerus fractures in the elderly, at a mean follow up of 35 months. Some 73 patients who had undergone reverse total shoulder arthroplasty for head-splitting three- or four-part fractures between October 2005 and October 2013 were identified, and of those 51 patients (with 52 treated shoulders) were included in the study. The study was performed in two orthopedic hospitals in Switzerland.

Patient demographics are summarized in Table 1. Some 19% patients sustained head-splitting fractures, 8% three-part, and 73% four-part fractures. Mean age of patients at the time of surgery was 77 years. The mean time from injury to having surgery was five days.

All procedures were performed using the Zimmer Reverse Anatomical Shoulder System, with a 0-200 stem retroversion to avoid tension on the greater tuberosity during internal rotation. Decision to cement the stem was made intra-operatively by the operating surgeon, depending on bone quality to press for the largest possible stem [18]. Greater tuberosity was always reattached where possible, in a transosseous manner using FibreWire. Remnants of unrepairable tuberosity were resected in four cases. Postoperative care included two suction drains for 48 h and sling for up to six weeks with passive rotation of shoulder and active-assisted elevation.

Constant score, SSV (Subjective Shoulder Value), age- and gender-matched relative CSAQ, pain level, and patient’s outcome satisfaction were recorded for 51 patients as the primary endpoints. Subsequently radiological studies were performed to assess any loosening, scapular notching, and position of the greater tuberosity. Some 20 patients were lost in the follow-up. Four patients died within the first year, unrelated to the diagnosis and procedure. Some 10 patients further died at a mean of 30 months postoperatively without having undergone any annual follow-up visits; therefore, they were excluded from the study. Five nursing home patients and one patient who moved country were contacted over phone, who all judged their results positively.

Results of the data collected are summarized in Table 2. The mean range of movement postoperatively showed 1180 FF, 1110 Abd., 180 external rotation, and 50 internal rotation. No loosening of the stem or glenoid component was identified radiologically. Notching was present in 33 patients (63%) and there were four patients (8%) with displaced tuberosities. There was no correlation of outcome with the amount of notching, however, patients with displaced or resected tuberosities had significantly lower CS and lesser ROM. Overall 92% of patients were rated Good or Excellent on the satisfaction scale.

Only revision rate was stated with regard to complications in the study. Four of 74 patients (5%) underwent revision surgery. One patient who required revision surgery developed a postoperative hematoma. One patient sustained a periprosthetic shaft fracture requiring revision with a long stem bypassing the humeral shaft fracture. Two patients developed deep infection of the joint, requiring staged revision process. One patient died from acute coronary syndrome four days after an uneventful surgery.

Other than having a relatively small number of patients with also a short follow-up period, the main limitation for this study was a high dropout rate (20 of 74 patients). However, authors did endeavor to determine the outcome of those through other means such as telephone conversations and reviewing medical notes. Authors conclude that RTSAAQ yield a very satisfactory outcome for elderly patients with osteoporotic bone who have sustained complex proximal humerus fractures. They also state that revision surgery may be required for those with displaced greater tuberosities following surgery, due to impaired functional outcome.

Reverse shoulder arthroplasty for comminuted proximal humerus fractures in the elderly patients has increased over the past several years, yet the published data evaluating the surgical outcome are limited. Most of the studies were relatively small, with short follow-up assessments. The current evidence regarding reverse shoulder arthroplasty for proximal humerus fractures is mostly retrospective case-series at Level IV evidence.

The primary purpose of this systematic review was to determine the impact of reverse shoulder arthroplasty on shoulder pain relief and function following complex three- and four-part proximal humerus fractures in elderly patients. The secondary objective was to assess clinical and radiological complications following this procedure for this indication.

All five studies agree that reverse shoulder arthroplasty provides excellent relief from pain. Study 1 reported a mean Constant pain score of 12.5 (out of 15), which is equivalent to 2.5 on the visual analog score. Study 2 demonstrated that the Bodily Pain component of the SF-36 was almost equal to that of the general U.S. population [15]. Study 3 supported this and reported a Constant pain score of 13 (equivalent to VAS of 3). Although Study 1 demonstrated good pain relief, the Constant score for pain was worse when compared to the contralateral shoulder taken as preoperative score before fracture. Study 3 reported that pain relief slowly deteriorated with time from one-year follow-up.

Good functional outcomes and range of movement (in terms of FF and Abd.) were reported in five studies. Study 1 reported a mean FF of 97° and Abd. of 86°; Study 2 reported 123° for FF and 113° for Abd.; and Study 3 reported a Constant score of 7.5 for FF (120° < FF < 150°) and Constant score of 6.5 for Abd. (120° < Abd < 150°) [16]. However, shoulder rotation remained limited. Study 1 attempted to repair the tuberosities at the time of operation which reported a mean external rotation of 30°, but failed to mention the outcome for internal rotation. Study 2 removed the tuberosities and reported 25° of external rotation and internal rotation to L4. Study 3 had removed remnants of the tuberosities, reported poor Constant scores for internal and external rotation. The results reported for anterior elevation and Abd. in these studies are superior to those reported for shoulder hemiarthroplasty [12]. Despite this, normal range of movement is not obtained, and restrictions in shoulder rotation have not been fully addressed. Simovitch et al. showed that fatty infiltration of the teres minor was associated with reduced postoperative external rotation [13]. Associated latissimus dorsi transfer [14]. and anatomic tuberosity reconstruction [15] may improve external rotation.

Study 1 reported a mean Constant score of 44, Study 2 as 67.9, and Study 3 as 53 points. These results are comparable to those published for reverse shoulder arthroplasty for cuff tear arthropathy [5, 8, 10- 12].

On radiological assessment, both Bufquin et al. and Klein et al. reported no evidence of any loosening in the humeral stem or the glenoid component at mean 22 and 33 months follow-up respectively [14-15]. Study 3 on the other hand reported some evidence of glenoid loosening in 63% of patients at a mean of 79.2 months follow-up. However, only one patient developed significant aseptic loosening after 12 years. This suggests that glenoid loosening is common and is likely to develop with time. This may result from the component being insecurely anchored, progressive glenoid bone loss, sub-optimal positioning (i.e. not seated low on glenoid) or secondary to trauma. The progressive deterioration in functional outcome with time noted in Study 3 may be due to aging of the patients, with gradual decreasing strength and activity; however, with the modified Constant score which accounts for this variable, the same effect was noted implying that this deterioration is not simply the result of aging. This progressive deterioration may be explained by the development of minimal amounts of loosening, which may be sufficient to modify the functional results.

Study 1 and Study 4 attempted to repair the tuberosities, whereas the other authors aimed to remove tuberosity remnants. Consequently, Study 1 reported 13.8% of tuberosity malunion and 38.1% of tuberosity nonunion. This may explain why this study had the lowest Constant score. However, Study 4 showed comparably better functional scores despite a tuberosity nonunion rate of 33%. Cazeneuve and Cristofari stated that impingement from tuberosity remnants resulted in three superior dislocations in their series [16]. Both Study 1 and Study 3 reported 25% scapular notching, whereas Study 2 reported only 5%. Scapular notching without loosening may contribute to glenoid migration. The cause of notching is not well established: latent sepsis [14], micro-movement of the lower screw [16], or impingement between the lateral border of the scapula and the medial border of the humeral cup during adduction may contribute [17]. The full significance of severe notching, however, remains unknown. However, Study 5 suggests this may not be functionally relevant in the elderly [16-20]. 

Reverse shoulder arthroplasty is a relatively new, unconventional approach to the treatment of a variety of difficult shoulder conditions in older individuals [19]. Thus, it is not surprising that it has been associated with frequent and substantial complications. Complication rates as high as 60% have been reported in Study 3 as 19.4% [17]. Only one intra-operative glenoid fracture was reported. There were two intraoperative humeral shaft fractures according to Study 4. Six patients out of a total of 178 patients in the five studies were dislocated (3.3%). The authors state that instability can be prevented by careful intra-operative examination to ensure full motion, proper version, absence of abutment, and no separation of the components when traction is applied to the humerus, combined with soft tissue repairs. Deep infection was reported in 3%, which may have occurred as a result of the hematoma formation, the magnitude of the surgery, and the compromised general health of the patients with these injuries. Neurological injuries occurred in 5% and chronic regional pain syndrome in 5%.

The limitations in the literature are substantial and primarily result from the limited number of published series, small number of patients, and short follow-up periods. Randomized control trials are missing. These studies have substantial variability for describing details of surgical treatment, measuring clinical outcomes, and reporting complications. Additionally, the surgical techniques utilized in the different studies vary in terms of the approach, prosthesis type, stem version, positioning of the glenoid component and whether the tuberosities were repaired or removed. This introduces limitations in making general conclusions because each surgical technique may have unique issues related to clinical outcomes and complications.

## Conclusions

The optimal management of complex three- and four-part displaced fractures of the proximal humerus in elderly patients with poor bone quality remains controversial. Our systematic review suggests that reverse shoulder arthroplasty is able to provide patients with good pain relief, function and range of FF and Abd. Restrictions in shoulder rotation are yet to be fully addressed. The temptation to offer this procedure needs to be balanced by an awareness of the considerable complication rates, cost, and potential for deteriorating function with time.

At present there are limited case-series studies available. We have applied strict eligibility criteria to the studies appraised to reduce the vast array of confounding factors that could affect the outcomes for this intervention. Large well-designed prospective randomized controlled trials with rigorous methodology are needed comparing the various treatment options for these patients, in order to ensure that these patients receive the best treatment available.
